# Moderate phenotype of a congenital myasthenic syndrome type 19 caused by mutation of the *COL13A1* gene: a case report

**DOI:** 10.1186/s13256-022-03268-z

**Published:** 2022-03-26

**Authors:** Mohamed Islam Kediha, Meriem Tazir, Damien Sternberg, Bruno Eymard, Lamia Alipacha

**Affiliations:** 1 Neurology Department, Mustapha Bacha University Hospital, Benyoucef Benkhedda University, Algiers, Algeria; 2 Myogenetics Laboratory, Pitié Salpetriére University Hospital, Paris, France; 3 Neurology Department, Pitié Salpetriére University Hospital, Paris, France

**Keywords:** Congenital, Phenotype, Gene, Col13A1, Myasthenia

## Abstract

**Background:**

Congenital myasthenic syndromes caused by mutations in the *COL13A1* gene are very rare and have a phenotype described as severe. We present the first case of congenital myasthenic syndrome described in Algeria and the Maghreb with a new mutation of this gene.

**Case presentation:**

We present an 8-year-old Algerian female patient, who presented with a moderate phenotype with bilateral ptosis that fluctuates during the day and has occurred since birth. During the investigation, and despite the very probable congenital origin, we ruled out other diagnoses that could induce pathology of the neuromuscular junction. The genetic study confirmed our diagnosis suspicion by highlighting a new mutation in the *COL13A1* gene.

**Conclusion:**

We report a case with a mutation of the *Col13A1* gene, reported in the Maghreb (North Africa), and whose phenotype is moderate compared with the majority of cases found in the literature.

## Background

The *COL13A1* gene is carried by the short arm of chromosome 10, encoding a protein (Collagen 13 with Alpha 1 chain), and was first described in 2015 [1]. It is implicated in a synaptic form of congenital myasthenic syndrome (CMS type 19 according to the new version of the Gene Table) [2]. The “Collagen 13 Alpha 1 protein” is a transmembrane protein composed of a short intracellular domain, a single transmembrane domain, and a collagen triple-helix ectodomain [3] [4]. The overall function of this protein is not well known, but a general role in connective tissue function has been suggested [5]. It is essential for the maturation of the neuromuscular junction, and development at the pre- and postsynaptic levels [6]. It also binds directly to the collagen tail of acetylcholinesterase and is believed to promote synaptic regeneration after peripheral nerve damage [7]. The general phenotype described for patients with mutations in the *COL13A1* gene is quite severe, with eating difficulties, breathing problems that can be serious, facial and axial weakness, and ptosis; however, few cases of ophthalmoplegia have been reported. Treatment with cholinesterase inhibitors appears to be moderately effective. However, actual conclusions on the phenotypic spectrum of this new gene remain limited given the very small number of patients described [8]. We report an Algerian family with an index case, and a similar case presenting a mutation in the *COL13A1* gene.

## Case presentation

We report on an 8-year-old Algerian female patient from a consanguineous marriage in Algeria, with a probably similar case (her maternal cousin, who has had bilateral ptosis since birth but whom we have not examined). Since birth, she has presented with drooping of the eyelids and the notion of double vision in the upwards gaze. These issues fluctuate during the day. Neonatal hypotonia was not noted, nor any delay in motor acquisitions. She has had several episodes of respiratory distress since the age of four, requiring bronchodilator treatment on demand, but has never been in intensive care unit. Functionally, she plays, runs, climbs, and goes down the stairs without difficulties. The baseline neurological examination (in 2018) found bilateral ptosis, a deficit in the orbicularis of the eyelids with limited upward eye movements. There was no dysmorphic syndrome and the prostigmine test was negative. Her myasthenia gravis score was 75/100 and her Quantitative Myasthenia Gravis (QMG) score was 8/39. The course was very stationary, not requiring treatment at this time. Her current myasthenic score is 85/100. Paraclinically, muscle enzymes and lactates were normal, as well as anti-acetylcholine receptor and anti-MuSK antibodies. The electromyography (EMG) examination found decrement in two nerve/muscle pairs (around 20%). The genetic study concluded a false sense mutation of exon 37 of the *COL13A1* gene (R675W mutation), a mutation that has not been reported in the literature. This is probably a new mutation of this gene, described for the first time in the Maghreb.

## Discussion

CMS linked to mutations in the *COL13A1* gene (CMS type 19) were described for the first time in three patients from two unrelated families [1], one of Indian origin and the other European. These three patients share the following clinical characteristics: onset at birth, slight facial dysmorphisms (ogival palate, low ear implantation, and micrognathia), net ptosis, and respiratory impairment (apnea and dyspnea). Two patients did not respond to pyridostigmine (PD) and one patient received a combination [3.4-diaminopyridine (3.4 DAP) and salbutamol]. Two mutations were identified: “c.1171 delG” and “c.523 delG.”

The second description (in 2019) [9] was of a 45-year-old Latin American patient, suffering since birth from a ptosis, dysphagia to solids, and muscular fatigue during effort, as well as difficulty in holding a discussion for more than 10 minutes. His disease progressed very slowly, with the onset of an episode of acute respiratory distress following cholecystectomy. There were no similar cases in the family and he was from a nonconsanguineous marriage but the parents were of the same ethnic origin. There was no dysmorphic syndrome. Low-frequency repetitive nerve stimulation (RNS) EMG showed marked decrement (15%, 50%, and 44%) in several nerve/muscle pairs. He also responded positively to treatment with PD. A new mutation in the *Col13A1* gene was identified (c.739C˃T).

A series of six patients (three European consanguineous families) was also published in 2019 [8]. In all of these patients, symptom onset was early (at birth) with a more severe phenotype: respiratory distress, dysphagia, hypotonia, and ptosis. Four patients presented with delayed walking. Five patients had significant scoliosis with facial dysmorphia (low ear implantation, arched palate, micrognathia). A net decrement was identified in all patients. The evolution was towards aggravation, because it was punctuated by severe respiratory and bulbar crises. PD-based treatment was offered to them with good clinical results. Three new mutations in the Col13A1 gene were identified.

The last and largest series was published in May 2019 [10]: 11 unrelated families including 7 consanguineous were studied. They had several ethnic origins (Brazil, Bangladesh, India, USA, Canada, Iran, Qatar, and West African but with a European ancestor). These 16 patients had some phenotypic homogeneity, with an onset at birth of ptosis, respiratory distress, and bulbar involvement. Ophthalmoplegia was less common. Respiratory attacks were seen in half of these patients. Dysmorphic syndrome was observed in 4 out of 16 patients, especially scoliosis. These disorders fluctuated more during childhood than adulthood in this series. PD treatment has given good results, but the combination of 3,4 DAP with salbutamol has markedly improved motor and respiratory function in some patients.

The clinical presentation of our patient seems slightly similar to the majority of cases described in the published series. She presented a ptosis that fluctuates during the day, whereas this fluctuation is not constant in the reported series.

Breathing difficulties remained minimal in our patient (no stay in intensive care), while severe respiratory distress has been described in the series by Dusl *et al*. [8] and Rodriguez-Cruz *et al*. [10]. We did not observe any facial dysmorphia or scoliosis in our patient, whereas the dysmorphic syndrome appears to be predominant in the patient series described above. The very slow evolution of our patient, whose parents currently refuse any treatment, seems to follow the case described by Marquardt in 2019, with a lighter phenotype where the patient did not request a neurological assessment until their mid-forties as the symptoms were so discreet. On the contrary, a patient in the series [1] died at the age of 8 from respiratory failure. Important differences in phenotypic severity were noticed in two patients from the same family in this series (intra- and interfamily phenotypic variability). Treatment with salbutamol alone has been tried in two patients from the same series (Dusl *et al*. [8]) with very good clinical improvement. This would suggest its indication, without associating it with 3.4 DAP.

## Conclusion

We report a rare case of CMS with a previously unidentified mutation of the *COL13A1* gene. This phenotype shares several characteristics with patients previously identified and described in other series. The genetic study and clinical examination of the affected cousin would help us better to understand this phenotype, which appears to be moderate. This result, the first in the Maghreb, and of which less than 30 cases have been published worldwide, further widens the genetic heterogeneity of CMS.

## Data Availability

Not applicable.

## Abbreviations

CMS: Congenital myasthenic syndromes
NMJ: Neuromuscular junction
QMJ: Quantitative myasthenia gravis
EMG: Electromyography
